# Paradoxical Effect of Pertussis Toxin on the Delayed Hypersensitivity Response to Autoantigens in Mice

**DOI:** 10.1371/journal.pone.0011983

**Published:** 2010-08-05

**Authors:** Rajwahrdhan Yadav, Sourojit Bhowmick, Philip Gorecki, James O'Rourke, Robert E. Cone

**Affiliations:** Department of Immunology, Connecticut Lions Vascular Vision Center, The University of Connecticut Health Center, Farmington, Connecticut, United States of America; New York University, United States of America

## Abstract

**Background:**

Pertussis toxin (PTX), an exotoxin of *Bordetella pertussis*, enhances the development of experimental autoimmune diseases such as experimental autoimmune uveitis (EAU) and experimental autoimmune encephalomyelitis (EAE) in rodent models. The mechanisms of the promotion of experimental autoimmune diseases by PTX may be based upon PTX-induced disruption of the blood eye/brain barriers facilitating the infiltration of inflammatory cells, the modulation of inflammatory cell migration and the enhancement of the activation of inflammatory cells. We hypothesized that the facilitation of experimental autoimmunity by PTX suggests that its influence on the *in vivo* immune response to auto-antigen may differ from its influence on non-self antigens.

**Methodology/Principal Findings:**

We have evaluated the effect of PTX on the simultaneous generation of delayed type hypersensitivity (DTH) responses and autoimmune responses to uveitogenic interphotoreceptor retinoid binding protein peptide (IRBP_161–180_), encephalitogenic myelin oligodendrocyte glycoprotein peptide (MOG_35–55_) or ovalbumin (OVA). PTX injection of mice immunized to IRBP peptide_161–180_ led to (i) the development of EAU as shown by histopathology of the retina, (ii) pro-inflammatory cytokine production by splenocytes in response to IRBP peptide _161–180_, and (iii) symptomatic EAE in mice immunized with encephalitogenic MOG peptide_35–55_. However, mice that received PTX had a reduced DTH response to IRBP_161–180_ peptide or MOG peptide_35–55_ when challenged distal to the site affected by autoreactive T cells. Moreover, footpad challenge with MOG_35–55_ peptide reduced EAE in mice immunized with MOG peptide. In contrast, the use of PTX when immunizing with OVA protein or an OVA immunogenic peptide did not affect the DTH response to OVA.

**Conclusions/Significance:**

The results suggest that that the reduced DTH response in mice receiving PTX may be specific for autoantigens and autoantigen-reactive T cells are diverted away from ectopic sites that received the autoantigen and towards the tissue site of the autoantigen.

## Introduction

Autoimmune uveitis, a disease that targets neural retina, is characterized by massive uveal inflammation, vasculitis, and the destruction of photoreceptor cells. Experimental autoimmune uveitis (EAU) is induced in mice by active immunization with evolutionarily conserved retinal proteins such as interphotoreceptor retinoid binding protein (IRBP), Complete Freunds' Adjuvant (CFA) and pertussis toxin [1]–[4]. Pertussis toxin (PTX), derived from *Bordatella perttussis* is usually required to induce experimental autoimmune disease. In addition to amplifying the activation of immunocompetent cells and proinflammatory cytokine production [5], [6]; PTX may play a role in opening the blood/brain barrier, and influences the migratory patterns of inflammatory cells [7]–[11]. In aggregate, PTX induces changes in vascular permeability, thus facilitating the breakdown of blood–tissue barriers and thereby facilitates the infiltration of inflammatory cells into the target organ.

Despite the use of PTX for over two decades to enhance the induction of experimental autoimmune disease, the mechanism of its action is still not yet understood. Although some of the enhancing effects of PTX on the induction of experimental autoimmunity are thought to be due to changes in vascular permeability when administered at the time of immunization, PTX also promotes the production of Th1 cytokines and low doses of PTX promote a delayed-type hypersensitivity response [10], [12]. The effects of PTX on antigen-presenting cells may underlie this phenomenon [13], [14]. Pertussis toxin also modulates the immune response to neural antigens injected with Incomplete Freund's Adjuvant: inducing Th1 cells and experimental autoimmune encephalomyelitis (EAE) in the presence of high frequencies of Th2 cells [14]. However, high doses of the toxin can result in reduced disease or DTH [5], [10]. The mechanism of this strict dose dependency of the effect of PTX is unknown.

Because PTX enhances proinflammatory cytokine production and Th1-based autoimmune diseases, we investigated the role of PTX in the induction of delayed-type hypersensitivity (DTH) to self-peptides in conjunction with the induction of autoimmune disease models. We observed that although PTX is critical for development of EAU in mice immunized with CFA, PTX and the uveitogenic peptide IRBP_161–180_, PTX reduced or retarded DTH responses to autoantigens in an antigen-specific manner by reducing cellular infiltration at the site of challenge even though PTX enhanced the production of proinflammatory cytokines. This inhibitory effect of PTX on DTH is limited to self antigens suggesting that the apparent inhibition of DTH to the autoantigens may be due to differential a migration of antigen specific lymphocytes and retention of effector T cells at a site expressing the self antigen.

## Materials and Methods

### Animals

Female C57BLB10.RIII-H2rH2-T18b (C57BLB10.RIII) and C57BL6 mice 6–8 weeks old were purchased from Jackson Laboratories (Bar Harbor ME and Charles River Laboratories (Wilmington, MA, USA) respectively. All animals were maintained by the Center for Laboratory Animal Care at the University of Connecticut Health Center. The use of animals adhered to the Association for Research in Vision and Ophthalmology (ARVO) resolution on the use of animals in ophthalmic and vision research. All work with animals has been reviewed and approved by the University of Connecticut Health Center Animal Care Committee (ACC 2004-380).

### Antigens and reagents

Human IRBP peptide_161–180_ (SGIPYIISYLHPGNTILHVD) that constitutes a major pathogenic epitope for C57BL10.RIII mice [4], [12] was prepared by Anaspec Laboratories, San Diego, CA. Myelin oligodendrocyte glycoprotein peptide_35–55_ (MOG_35–55_) was prepared by the Yale University W.M. Keck Facility. Ovalbumin (OVA) peptide; (TEWTSSNVMEERKIKV amino acids 265–280) for CD4 T cells purchased from Invitrogen Life Sciences (Carlsbad, CA) was the generous gift of Drs. A. Vella and A. Menoret, Department of Immunology, University of Connecticut Health Center. Ovalbumin protein was purchased from Sigma (St. Louis, MO). Pertussis toxin (PTX) was purchased from List Biological Laboratories, Campbell,CA. Complete Freund's adjuvant (CFA),Incomplete Freund's Adjuvant (IFA) and *Mycobacterium tuberculosis* strain H37RA were purchased from Difco (Detroit, MI).

### Induction and scoring of EAU [15]

Mice were immunized on the dorsal surface of their back intradermally with a 0.2 ml of the emulsion of 50–100 µg IRBP_161–180_ peptide in CFA (1∶1, v/v), containing 2.5 mg/ml of M. tuberculosis H37RA. Mice were injected with PTX, 1 µg in phosphate-buffered saline (PBS, pH 7.2) in a total volume of 0.1 ml i.p. 24 h after immunization with IRBP and CFA. Freshly enucleated eyes were collected for histopathology on specific days after immunization, and were fixed in 4% paraformaldehyde (PFA) solution. The incidence and severity of EAU was scored on an arbitrary scale of 0–4 on hematoxylin and eosin-stained sections, according to a semi quantitative system described [15]. Two independent observers did the scoring in blinded manner and results compared. The clinical scale measured was as follows: 0  =  normal, 1  =  mild distortion of layers of retina, 2  =  mild to moderate distortion of layers of retina and minimal inflammatory cell infiltrate, 3  =  moderate distortion of layers of retina and massive inflammatory cell infiltrate and 4  =  severe distortion of retinal layers with total disruption of retinal architecture and massive cellular infiltrate.

### Induction and scoring of Experimental Allergic Encephalomyelitis

For the induction of active EAE, C57BL/6 mice were injected intradermally (ID) with 200 µg of emulsion containing 200 µg MOG_35–55_ in Incomplete Freund's Adjuvant (IFA) supplemented with 500 µg of *M. tuberculosis*. Mice were injected i.p with 200 ng PTX in 100 µl of PBS shortly after and 48 h after the first immunization. Following immunization, animals were kept under observation to score the disease. The study was done in a blinded fashion. The clinical scale measured was as follows: 0  =  normal, 1  =  limp tail, 2  =  paraparesis with a clumsy gait, 3  =  hind limb paralysis, 4  =  quadriplegia, 5  =  death.

### Induction of Delayed-Type Hypersensitivity

To induce a maximum DTH response mice were immunized with a subcutaneous (sc) injection of 50 µl CFA containing 100 µg emulsified IRBP peptide_161–180_, or 200µg MOG peptide_35–55_, 50 µg OVA_265-280_ peptide or 200 µg OVA protein into a flank. The control group did not receive any immunization or in some cases CFA without IRBP, MOG peptide or OVA. Seven days after immunization the mice were anesthetized with ketamine (75 mg/kg) and xylazine (15 mg/kg) and footpad thickness of both hind footpads measured with a digital micrometer (Mitatoyo, Tokyo, Japan). One footpad was challenged with an intradermal (ID) injection of 50 µg IRBP_161-180_ peptide, MOG_35-55_ peptide, OVA or OVA_265-280_ peptide in PBS and the other footpad was challenged with PBS only. Approximately 24, 48 and sometimes 72 h later, the mice were anesthetized with ketamine/xylazine and footpad thickness re-measured. Swelling was computed as the difference in thickness (in µm) of the challenged footpad at 24 h minus the difference in thickness (in µm) of the vehicle-challenged footpad at 24 h. Some mice were euthanized and footpads were collected and fixed in 4% paraformaldehyde (PFA). Histopathology sections were taken and stained with hematoxylin and eosin (H&E). Sections were analyzed for leukocyte infiltration and inflammatory cell accumulation.

### *In Vitro* Stimulation of T cells with IRBP peptide

Splenocytes were obtained from immunized mice on day 7-post immunization. Splenocytes were cultured at 37°C in flat bottom 12 well plates at a concentration of 1×10^7^ cells/well and stimulated with 100 ng IRBP peptide or medium only in a volume of 0.5 ml. Supernatants were harvested 24 h post in-vitro culture and stored at −20°C. until assayed for cytokines.

### Cytokine assays

Supernatants that were collected after 24 h from in-vitro cultures were assayed for presence of cytokines (IL-1ß, TNF-α, IFN-γ and IL-10) by ELISA using kits from R & D Laboratories (Minneapolis,MN) as described by the manufacturer.

### Statistics

Footpad swelling and cytokine assays were compared between groups and a paired t-test was used to do statistical analysis. In all comparisons, P<0.05 was used to determine statistical significance.

## Results

### Induction of EAU by IRBP peptide_161–180_

**Figure 1:**
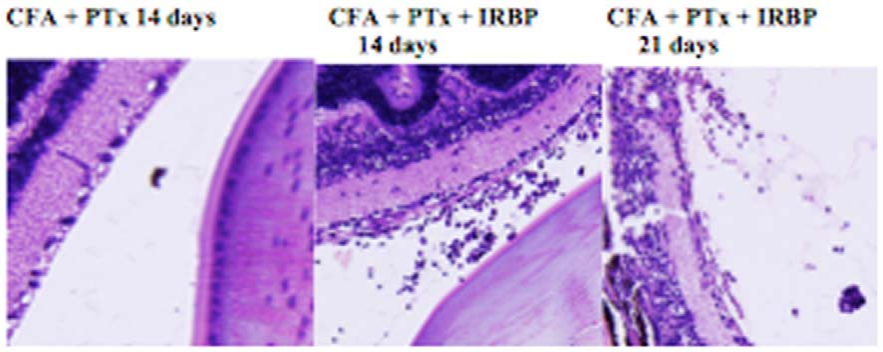
Induction of EAU with IRBP peptide. C57BL10.RIII mice received sc IRBP peptide_161–180_,+ CFA. Twenty-four hr later the mice received PTX i.p. Fourteen or 21 days P.I. the mice were euthanized, eyes enucleated, sectioned and stained with H&E. Figure is representative of sections from 5 individual mice.

**Figure 2:**
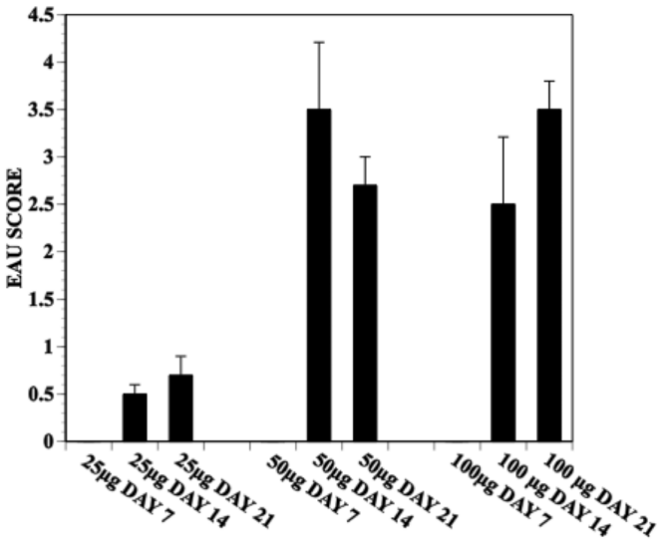
Dose/response and kinetics of the development of EAU by immunization with IRBP peptide. Mice were immunized with IRBP peptide_161–180_, CFA and PTX. Mice were euthanized 7,14 or 21 days P.I., eyes enucleated and sectioned and stained with H&E. EAU is scored as described in Materials and Methods and the data represent the average score +/−S.E.M. of 5–6 mice/group.

C57BL10.RIII mice were immunized with IRBP peptide_161–180_, CFA and PTX and euthanized on days 14 or 21 PI. The eyes were enucleated; sections obtained and stained with H&E. Sections were analyzed for integrity, architecture of the retinal layer and mononuclear cell infiltrate. The histopathology of the eyes of mice receiving CFA + PTX only, CFA or PTX only, or CFA + IRBP peptide but no PTX were no different from mice receiving PBS only or no injection. Mice that received 50–100 µg IRBP peptide, CFA and 1 µg PTX attained a loss of architecture of the retinal layers and extensive mononuclear cell infiltration 14 days P.I. (Fig 1). The histopathology of the retinal layer was similar on day 14 P.I. in mice immunized with 50 or 100 µg IRBP peptide. Extensive mononuclear cell infiltration and a marked disorder of the retinal outer nuclear layer (EAU score 3+) was observed by day 14 or 21 in mice receiving 50 or 100 µg IRBP peptide and 0.5–1 µg PTX. EAU was diminished significantly by day 21 P.I. in mice immunized with 25 or 50 mg IRBP peptide but was maintained in mice receiving 100 µg IRBP peptide (Fig. 2). Although damage to the outer nuclear layer of the retina was extensive by day 21 P.I., the infiltrate of mononuclear cells was diminished.

### PTX enhances Th1 cytokine production but diminishes the DTH response to IRBP peptide_161–180_

#### Cytokine production

**Figure 3:**
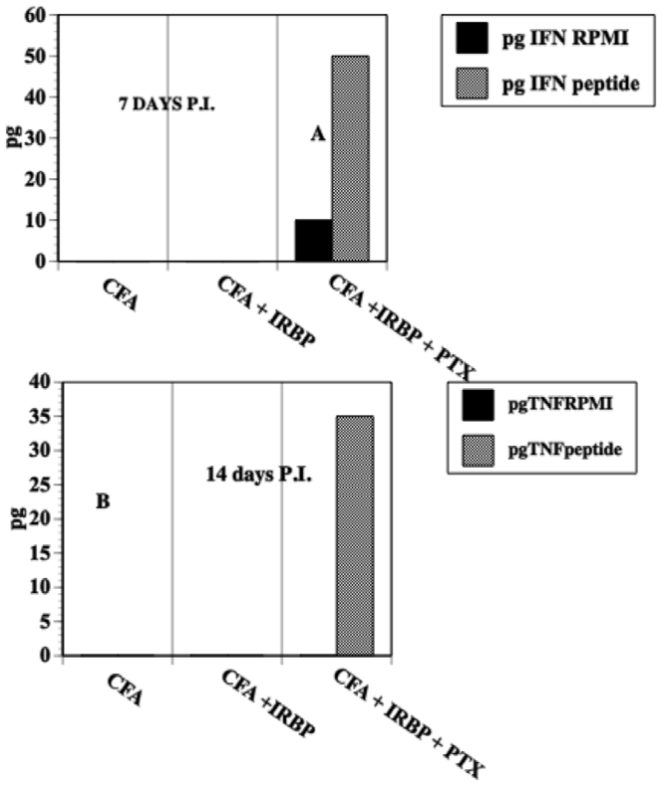
PTX potentiates the production of Th1 cytokines by splenocytes. C57BL10.RIII mice were immunized with IRBP peptide_161–180_, CFA +/− PTX. Splenocytes were obtained from immunized mice on day 7 or 14 days P.I. Splenocytes were cultured at 37°C. in flat bottom 12 well plates at a concentration of 1×10^7^ cells/well and stimulated with 100 µg IRBP peptide or medium only in a volume of 0.5 ml. Supernatants from 3 cultures were harvested 24 h post in-vitro culture and stored at −20°C. Supernatants were assayed by ELISA for TNF-α or IFN-γ ~.

Fourteen days after C57BL10.RIII mice were immunized with IRBP peptide_161–180_, spleens were recovered and spleen cells cultured +/− the IRBP peptide. Splenocytes recovered from mice immunized with CFA, IRBP and PTX produced measurable amounts of TNF-α and IFN-γ when cultured *in vitro* without IRBP peptide (Figure 3A,B). However, mice receiving PTX produced 5-35-fold more TNF-α or IFN-γ. More cytokines were produced when the splenocytes were cultured with lipopolysaccharide (LPS) and IRBP peptide (data not shown).

#### Delayed-type Hypersensitivity

**Figure 4:**
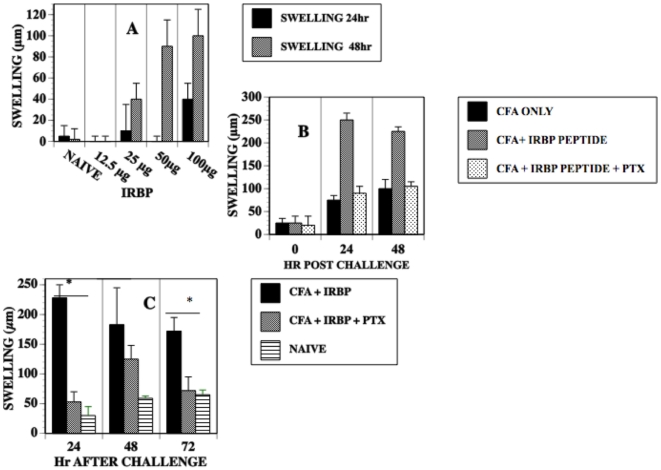
DTH to IRBP peptide_161–180_ is diminished in mice receiving PTX. C57BL10.RIII mice were immunized with IRBP peptide, CFA +/− PTX. Seven days P.I. a footpad was challenged with an intradermal injection of IRBP peptide and swelling measured 24 and 48 hr after challenge. The data represents the footpad swelling +/− S.E.M. of 6–8 mice/group in two experiments.

**Figure 5:**
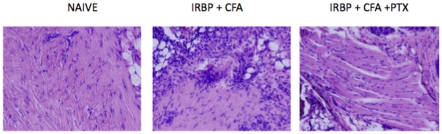
Pertussis toxin reduces the cellular infiltrate in the DTH response to IRBP peptide_161–180_. C57BL10.RIII mice were immunized with IRBP peptide _161–180_, CFA +/− PTX. Seven days P.I. a footpad was challenged with intradermal IRBP peptide_161–180_. Twenty four hr after challenge footpads were harvested, sectioned and stained with H&E. Section is 10X and represents footpad of one of three mice.

Because PTX augments the generation of EAU and the production of inflammatory cytokines, we reasoned that PTX would also enhance a DTH response to IRBP peptide. To test this hypothesis, mice were immunized with IRBP peptide, CFA and varying doses of PTX. Seven days P.I. a footpad of the immunized mice was challenged with an intradermal injection of IRBP peptide. Footpad thickness was measured pre, 24, and 48 hr post challenge. Although mice immunized with 12.5, 25 µg IRBP peptide and CFA produced footpad swelling approximately 50% greater than naïve mice when challenged with IRBP peptide (data not shown) maximum swelling was achieved in mice immunized with 100 µg IRBP peptide and CFA (Figure 4). However mice receiving 1 and 5 µg PTX post immunization with IRBP peptide and CFA had markedly reduced swelling after challenge. In many instances this reduction in swelling in mice receiving PTX was noted at 24 hours post challenge but sometimes was less profound at 48 hr post challenge. Seventy-two hr post challenge the increment in swelling in mice treated with PTX was no greater than that of naïve mice. There was no effect on DTH swelling in mice injected with less than 250 ng PTX (data not shown). Thus, there is a dichotomy in proinflammatory cytokine production by splenocytes and DTH responses in the periphery. H & E sections of the challenged footpads (Fig 5) revealed a reduced mononuclear cell infiltrate in the challenged footpad of immunized mice that received PTX.

### PTX fails to diminish the DTH response to OVA but reduces the DTH response to MOG_35–55_ peptide

**Figure 6:**
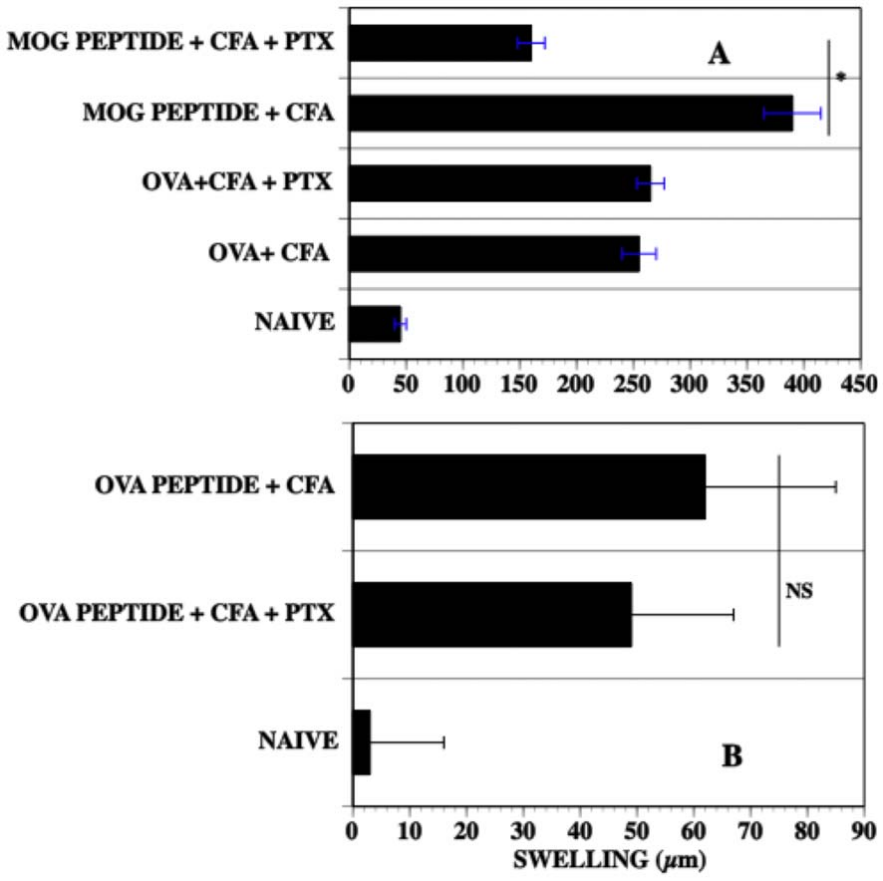
PTX reduces DTH to MOG_35–**55**_ **peptide but not to OVA.** (A) C57BL/6 mice were immunized with MOG _35–55_ peptide or OVA, CFA +/− PTX. Seven days P.I. footpads of the mice were challenged with intradermal MOG peptide_35–55_ or OVA respectively. Swelling was measured 24 and 48 hr later. Data represents the mean swelling (µm) +/−S.E.M. of five mice/group. The experiment was done twice. (B) C57BL10.RIII mice were immunized with OVA_265–280_ peptide, CFA +/− pertussis toxin. Seven days P.I, footpads were challenged with 50 µg OVA_35–55_ peptide and swelling measured 24 hr post-challenge.Data represents the mean +/−S.E.M. of 4 mice. NS: not significant.

**Figure 7:**
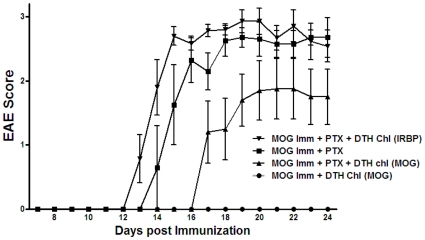
EAE is reduced by a challenge to the footpad with MOG_35–55_ peptide. EAE was assessed fourteen days after C57BL/6 mice were immunized with MOG peptide_35**–**55_, CFA and PTX or immunized and a footpad challenged with intradermal MOG peptide_35**–**55_ or IRBP peptide_161**–**180_ 7 days P.I. Following immunization, animals were kept under observation to score the disease. The study was done in a blinded fashion. The EAE scale was as follows: 0  =  normal, 1  =  limp tail, 2  =  paraparesis with a clumsy gait, 3  =  hind limb paralysis, 4  =  quadriplegia, 5  =  death. Data represents the mean score +/− S.E.M. of five mice/point. The experiment was done twice. DTH: delayed type hypersensitivity, chl: challenge

We investigated the effect of PTX on immunization for a local DTH response to OVA protein, OVA_265–280_ peptide or the encephalitogenic MOG peptide_35–55_ by immunizing C57BL/6 mice with MOG peptide, OVA protein, OVA_265–280_ peptide, CFA +/− PTX. Seven days after immunization, a footpad of the immunized mice was challenged with intradermal OVA_265–280_ peptide or MOG_35–55_ peptide. Footpad swelling to a challenge with OVA protein or OVA peptide was not affected by the administration of PTX during immunization (Fig 6A,B). In contrast, 7 days P.I. in mice immunized with encephalitogenic MOG_35–55_ peptide CFA and PTX, the local DTH response to MOG_35–55_ peptide was reduced significantly as compared to mice immunized with MOG_35–55_ peptide and CFA only. However, mice receiving PTX with MOG_35–55_ peptide PTX and CFA had robust EAE 14 days P.I. (Fig 7). However, EAE was reduced at day 14 in MOG_35–55_ peptide-immunized mice whose footpad received intradermal MOG peptide on day 7 P.I. EAE was not affected in MOG peptide immunized whose footpad was challenged with IRBP peptide.

## Discussion

The induction of EAU in C57BL10.RIII mice by immunization with IRBP peptide_161**–**180_, CFA and PTX is well documented [1]–[5], [12], [15]. Consistent with those reports we observed that EAU was not induced unless the mice received PTX post- immunization with IRBP peptide and CFA. EAU was characterized by a strong infiltration of mononuclear cells to the retina and extensive damage to the retinal outer nuclear layer. By day 21, damage to the outer nuclear layer was equal to or greater than that observed 14 days P.I. but more than 21 days P.I., the number of infiltrated mononuclear cells was reduced markedly or not detected. Additionally, 21+ days P.I. there appeared to be some resolution of damage to the outer nuclear layer of the retina (data not shown).

PTX enhanced the production of the Th1 cytokines IFN-γ and TNF-α7 days P.I. consistent with the effect of PTX on the enhancement of the induction of cell-mediated immunity [5], [6], [10], [12]. In fact, Th17 cells, essential to autoimmunity and DTH [16]–[18] are promoted by PTX [19]. Accordingly, we reasoned that PTX would also enhance a DTH response in the C57BL10.RIII mice immunized with CFA and IRBP. To our surprise, we found that the DTH-induced swelling of footpads of IRBP immunized mice challenged with intradermal IRBP was absent or reduced markedly in mice receiving PTX. This swelling correlated with a reduced number of monocytic cells that infiltrated the site challenged with IRBP peptide_161–180_. Our results with IRBP peptide differ from those of Silver *et al* [12] who demonstrated an enhancement of DTH to IRBP protein in C57BL10.RIII mice receiving PTX. However, Silver *et al* measured DTH to IRBP -induced DTH on day 21 P.I. rather than day 7 P.I. using significantly lower amounts of PTX. Although we observed lower DTH on day 21 P.I., 24 hr after challenge, there was a modest reduction in swelling in the footpads of mice immunized with IRBP peptide and CFA only and some increase in swelling in mice receiving PTX (Fig 4). However, the increase in swelling in the group receiving PTX was transient and not apparent 72 hr after challenge. Although we observed extensive damage to the outer nuclear layer by day 21 P.I., monocytic infiltration to the retina was reduced markedly. Silver *et al* did not get robust DTH without PTX although in our hands mice immunized with MOG_161–180_ peptide and CFA (without PTX) did develop robust DTH. Additionally, the enhancement of DTH by PTX is due to the B subunit while the A subunit may induce the inhibition of an autoimmune response, or, at the doses of PTX we used, the inhibition of DTH [5].

Although the DTH response of C57BL/6 mice to OVA or OVA peptide was not influenced by PTX, the DTH response of C57BL/6 mice to encephalitogenic MOG_35–55_ peptide was diminished in mice immunized with MOG_35–55_ peptide, CFA and PTX even though these mice exhibited robust EAE. However, EAE was significantly reduced in these mice after their footpad was challenged with MOG peptide but not with IRBP peptide _161–180_.

Thus, mice immunized with IRBP or MOG (self) peptide, CFA and a PTX dose that facilitates the induction of autoimmunity had a strong immune response to these autoantigens yet exhibited a diminished or absent DTH reaction when challenged with the autopeptides. In contrast, immunized mice that did not receive PTX (that did not develop autoimmunity) have a strong DTH response to the self-peptides. Moreover, the DTH response to OVA was not affected by PTX. In fact, PTX can amplify the DTH response to bacterial antigens [20]. Although PTX is thought to modulate lymphocyte migration [12], [21], the administration of PTX before sensitized mice are challenged with the cognate antigen does not inhibit the DTH reaction [21]. Therefore, it is unlikely that PTX administered 6–10 days before the challenge affected the DTH response by activated T cells that includes the recruitment of monocytic cells. In aggregate, because the suppression of DTH by PTX is localized to self antigens, it is tempting to speculate that the lack of a local DTH response in mice with a localized, ongoing autoimmune response is due to a “diversion” of sensitized T cells to the site(s) containing the autoantigen, eg the retina (IRBP) or neurons (MOG). In that regard, the apparent reduction in EAE in MOG-immunized mice that received a challenge to the footpad with MOG peptide could be due to a diversion of antigen-reactive T cells to the challenge site. These possibilities are under investigation.
